# Epidemiology of voluntarily reported dog and cat bite incidents in a large-scale, multi-site veterinary network in the UK and Ireland

**DOI:** 10.3389/fvets.2026.1916141

**Published:** 2026-10-06

**Authors:** Rachel Malkani, David Singleton, David Martin, Marian Vinke, Laura Playforth, Michaela Collis, James A. Oxley

**Affiliations:** IVC Evidensia, Bristol, United Kingdom

**Keywords:** bite, cat, dog, injury, safety, veterinary

## Abstract

**Introduction:**

Dog and cat bites are an occupational risk within veterinary practise. Despite this, there is minimal research exploring the occurrence and details surrounding such incidents.

**Methods:**

The aim of this study was to use a large veterinary group’s existing incident and near miss reporting system in the UK to explore the incidence of dog and cat bite reporting and surrounding details of such incidents.

**Results:**

Dog and cat bite incidents were identified from January 2024 to October 2025 covering all clinics in the UK and Ireland (>1,000). A total of 2,327 dog (1,239; 53.24%) and cat (1,088; 46.76%) bites were reported. The most common anatomical bite location was reported to be the hands and fingers for both dog (63.0%) and cat (80.6%) bites. The majority of bites for cats (77.96%) and dogs (80.60%) occurred between 9 a.m. and 6 p.m. Location of in-clinic bites differed between staff, with the veterinarians most commonly bitten in the consult room and non-veterinarians most often bitten in the preparation area. Regarding perceived behaviour, the term ‘aggression’ was most frequently used in reports.

**Conclusion:**

In conclusion, this report highlights the common occurrence of dog and cat bite related injuries, providing insights into surrounding factors. Factors need to be considered to reduce bites such as low stress handling, environment adaption and encouraging advancement of knowledge of animal behaviour. More needs to be done to help the reporting of incidents, incorporate human behaviour change approach and education to prevent bite incidents.

## 1 Introduction

Injuries within the veterinary profession are commonplace to the extent that such incidents are often regarded by veterinary staff as the *norm,* expected, or a ‘part of the job’ (1–4). Of the animal related injuries to veterinary staff, dog and cat bites are consistently reported as the most common across multiple countries [e.g., UK (4), US (5), Australia (6–8)]. In the UK, Tulloch et al. (4) surveyed 647 veterinary professionals from a large chain of veterinary clinics and found that the most common recent and severe animal injuries experienced by both veterinary nurses and veterinary surgeons were dog and cat bites. Despite this, more recently, Tulloch et al. (9) audited a large veterinary chain’s accident reporting system and found that in the UK that most injuries (69 for every 100) went unreported, highlighting the need for further research, education, behaviour change and training. This underreporting suggests that the true bite frequency and characteristics of injuries may be incompletely understood.

The direct impact of dog bite injuries, depending on the definition or perception and context of a bite/injury (3, 10, 11), can vary from minor injury (bruising to the skin) to serious or fatal injuries (12, 13). Dog bite injuries to veterinary team members can also result in time off work, loss of income, legal implications, and/or effect on mental health (e.g., trauma, loss of confidence, increased anxiety/fear) (4, 14). These impacts highlight the importance of understanding not only the occurrence of bite incidents, but also the context in which they arise.

Various animal, procedural and human factors are likely to increase bite injury risk to veterinary team members within the clinic. Firstly, a dog may attend in a fearful or painful state even before entering the consultation room (11). Whilst in the consultation room, a close (e.g., physical checkup, handling/restraint) and/or invasive procedure (e.g., blood draw, anaesthesia, muzzling) may be needed, causing fear, stress and/or pain, potentially further increasing bite injury risk (11). Furthermore, staff with less experience of animals have been found to be at increased risk of dog bite and animal-related injury (7, 15).

A range of measures can be taken to help mitigate the risk of dog and cat bite injuries, such as improvement in understanding of animal behaviour and body language, ensuring reduced environmental stressors in waiting rooms and consultation rooms (such as noise, odours, flooring etc.), avoiding close intraspecific animal interactions in the waiting rooms (e.g., design), and using appropriate handling/restraint methods, including protection equipment such as muzzles and wraps (16). However, with considerable individual variation between each animal, and without the ability to directly determine their experience, it is difficult to identify a single cause for a bite. Instead, the cumulative impact of multiple stressors is likely important, with thresholds for biting varying between context and arousal levels.

Despite the frequency of dog and cat bites in veterinary practise, there is limited research focusing solely on dog and cat bite occurrence within the veterinary profession.

Therefore, the authors aimed to review dog and cat bites reports submitted by veterinary team members that have been collated through a large veterinary chains incident and near miss reporting system. More specifically, using both quantitative and qualitative methods, we aimed to understand the team member role involved, time and location of the incident, procedure being undertaken during the incident, and the language used to describe the behaviour of the animal by team members. Finally, based on the findings, the authors also recommend future changes to the reporting system to improve reporting, and discuss additional information to encourage the reporting of injuries at clinic level. Additionally, we also make recommendations about the need for the advancement of knowledge in animal behaviour, low stress handling methods and adaptation to the environment(s).

## 2 Materials and methods

### 2.1 Injury data

Anonymised incident report data from the ‘UK and Ireland’ region were downloaded from the veterinary chain’s reporting site ‘Assure’ (Version: 36.2.473.0) from 01/01/2024 to 28/10/2025 708 clinics reported bite incidents. Out of a total of 2,860 reports, 2,545 reported their clinic name. The incident report questions can be found in Appendix 1. It should be noted that not all questions are mandatory to be completed. Data cleaning were conducted in R (version 4.2.2). Duplicate records were removed and records with missing or empty values were excluded on a question-by-question basis. Incident times were converted into standard date-time. For analysis of incident location and staff role, categories were consolidated into relevant groups. Descriptive statistics including the total number of bite incidents, what part of the body was affected from a bite, injury type, time of bites, and job role were undertaken using the “dplyr,” “stringr,” and “gt” package.

### 2.2 Data analysis

To analyse the written descriptions of bite incidents, data were filtered by species into cats and dogs; free-text responses were extracted from the survey field “Describe the incident as fully as possible in your own words”. Common stop words (e.g., “the,” “and,” “of”) were removed to reduce noise. Word frequencies were summarised using the R Studio package “dplyr” to summarise the most commonly used words and bigrams. In addition to describing the incident, the authors also analysed free-text responses describing the type of work being carried out at the time of each bite incident, taking the same approach as described above.

In the present study we do not define a bite and include all reported bite incidents based on self-reported identification of a bite having taken place.

To explore any perceived emotions or states of the animal during the bite incident, we manually screened the free text column describing the bite incident and summarised any emotional or attributable terms associated with the animal. These were “grumpy,” “angry,” “aggressive,” “agitated,” “fearful,” “nervous,” “scared,” “anxious,” “panicked,” “calm,” “relaxed,” “conflict,” “defensive,” “protective,” “guarding” and “possessive.” Using the “tidytext” package, words matching the predefined emotional terms were identified and the frequency of each term was calculated.

Spearman’s rank correlation was used to assess the relationship between the number of registered cats and dogs per clinic and the corresponding number of bite incidents. To examine whether the location of bite incidents differed between job roles, chi-squared tests were used. The analysis included only clinical staff (veterinary surgeons and veterinary nurses) and incidents that occurred in the consult room, kennel area, theatre/surgical area, or preparation area. Throughout this analysis, statistical significance was defined as *p* < 0.05 and for visualisations, the R package “ggplot2” was used (17).

## 3 Results

There were 2,420 bites recorded across the ‘UK and Ireland’ region clinics between 01/01/2024 and 28/10/2025.

The majority of bites were from cats and dogs (Table 1).

**Table 1:** Total number of bite incidents in 2024 and up to October 2025.

Species	Bite count	Percentage
Cat	1,239	51.20
Dog	1,088	44.96
Rodent	37	1.53
Other	24	0.99
Bird	15	0.62
Exotic (not native to country)	7	0.29
Horse	7	0.29
Rabbit	2	0.08
Donkey	1	0.04

A total of 2,327 dog and cat bites were reported.

### 3.1 Anatomical location of the bite

Where the affected body part was recorded, cat bite incidents (*n* = 433) predominantly involved the hands and fingers, which together represented more than 80% of all affected body areas. Similarly, most dog bites (*n* = 384) involved the hands and fingers, accounting for 63% of incidents; bites to the arm represented 18% of incidents (Appendix 2).

### 3.2 Description of incident

Cat bite incidents were most commonly described during the handling and restraint of cats, with hands and fingers being the body parts most frequently affected. Dog bite incidents also most frequently involved injuries to the hands and fingers and were commonly described as occurring when dogs lunged or reacted during handling (Figure 1).

**Figure 1:**
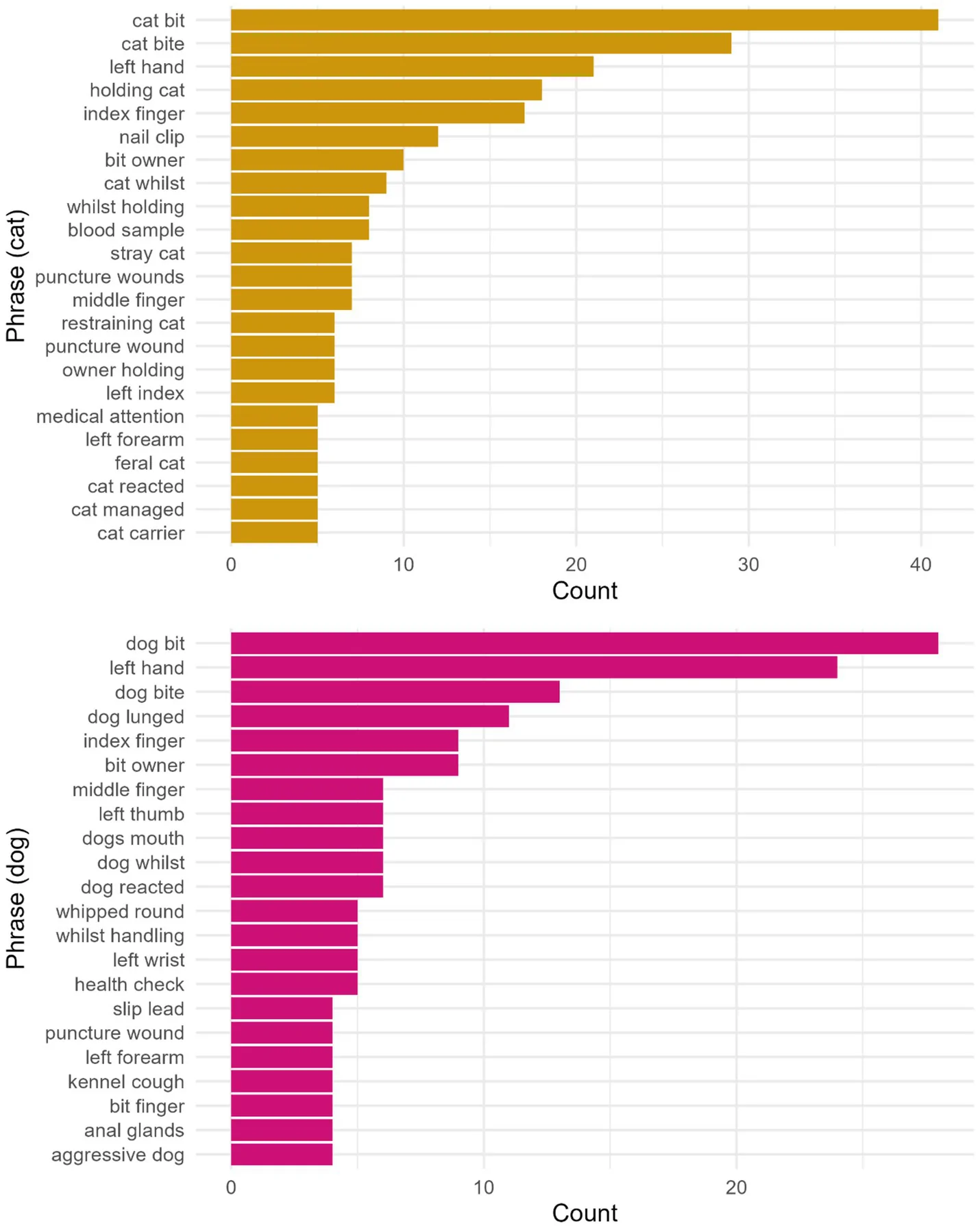
Most common bigrams used when describing a bite incident [orange = cats (*n* = 962), pink = dogs (*n* = 823)].

### 3.3 Description of work being undertaken

When describing the work being carried out at the time of the bite incident, for cats (provided in 303 records), bite incidents were most frequently reported during restraint and handling activities, including holding, handling and restraining the cat (*n* = 27). Other commonly reported procedures included nail clipping (*n* = 13), blood sampling and similar (*n* = 21), and clinical examinations (*n* = 17) (Figure 2). The most commonly reported procedures for dogs (in 258 records) included health checks (*n* = 9), clinical examinations (*n* = 6), and blood sampling (*n* = 5), alongside handling and restraint related activities (*n* = 9) (Figure 2).

**Figure 2:**
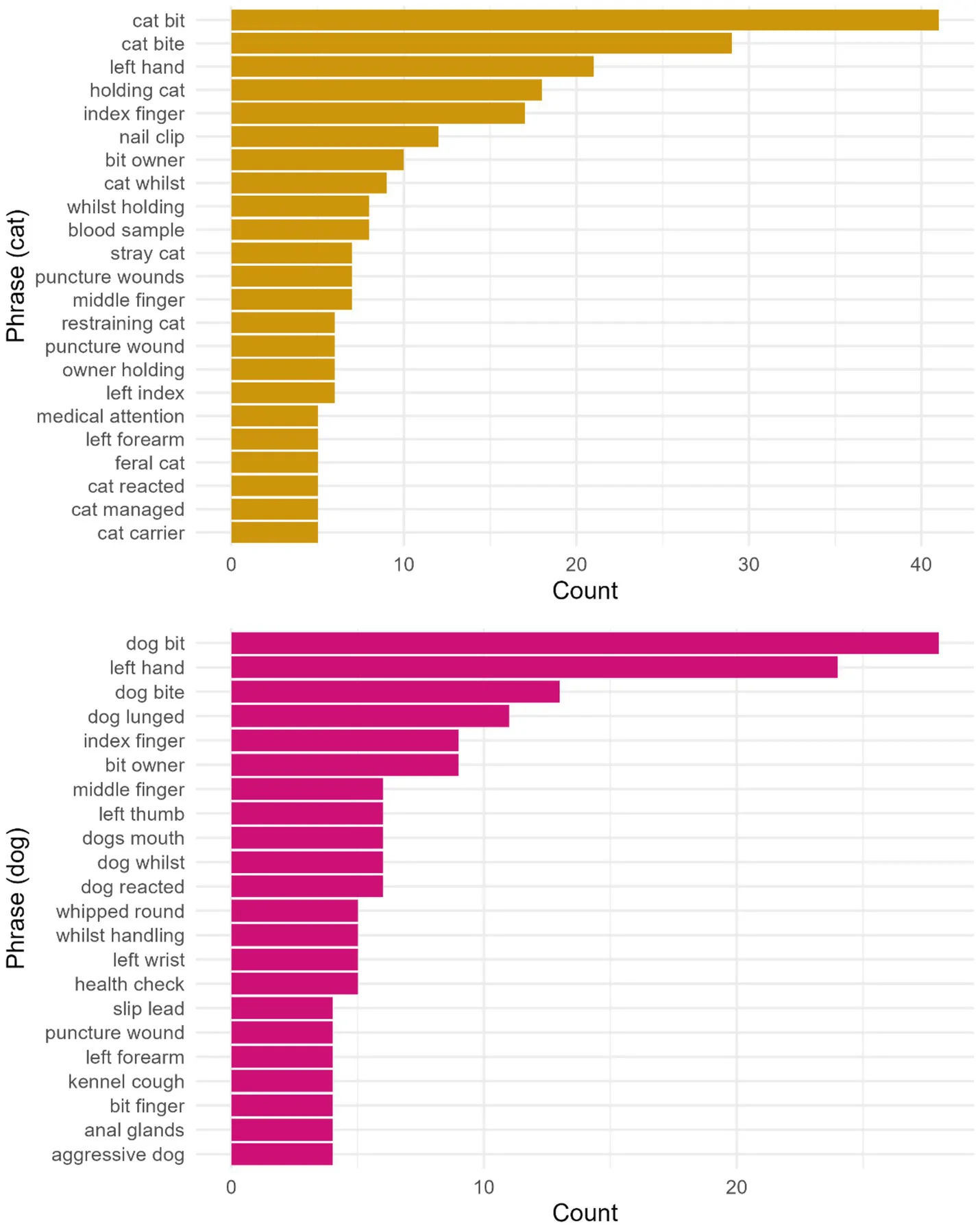
Most common bigrams used when describing the work being carried out during the bite incident [orange = cats (*n* = 1,057), pink = dogs (*n* = 907)].

### 3.4 Emotional words attributed to the animal

Cats (*n* = 137) and dogs (*n* = 129) were most commonly described as being aggressive during the bite incident. The terms scared and panicked followed aggressive in cats; whereas, nervous and anxious were more common words used to describe dogs (Figure 3).

**Figure 3:**
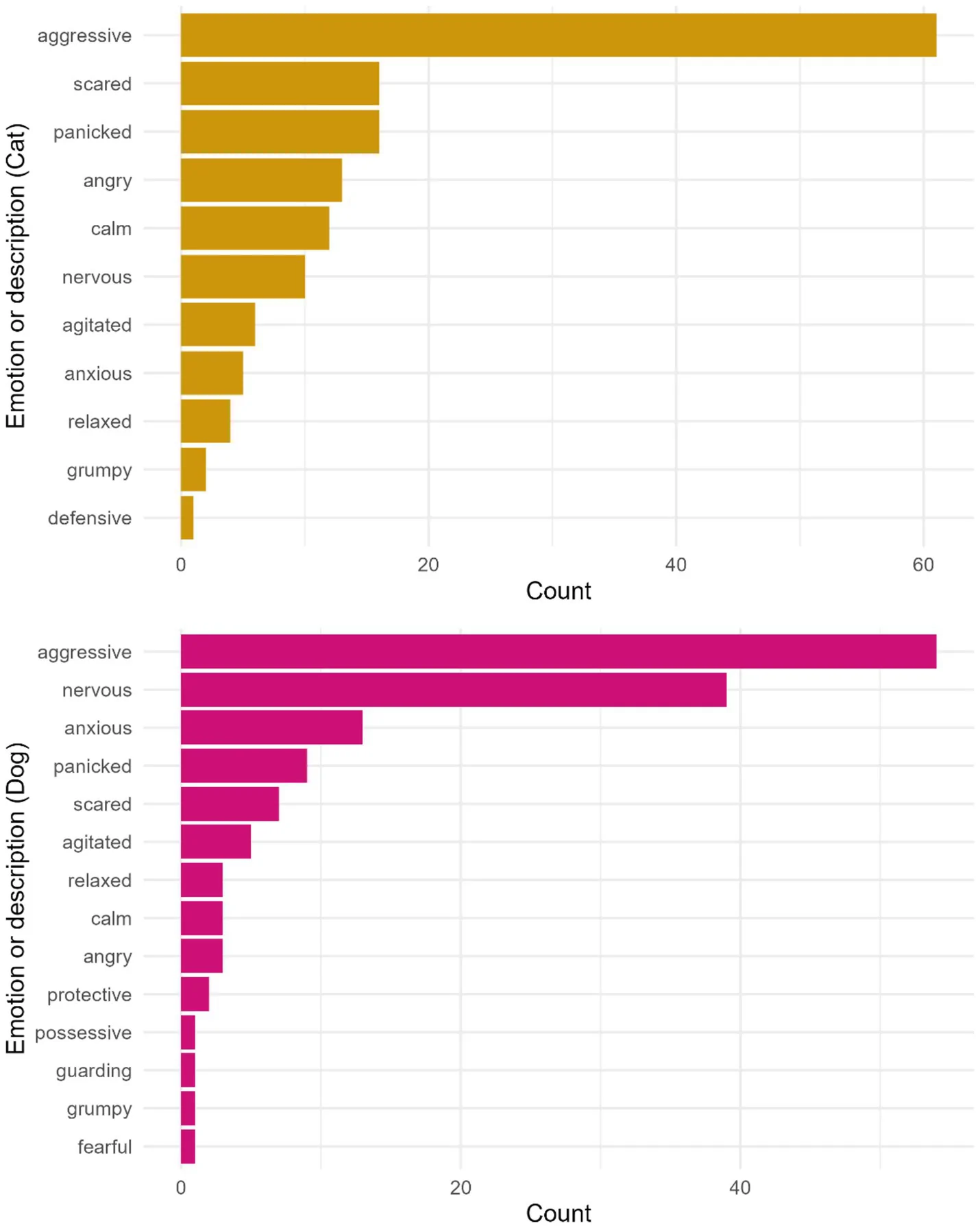
Frequency of emotional terms used to describe the dog or cat during the bite incident [orange = cats (*n* = 137), pink = dogs (*n* = 129)].

### 3.5 Time of bite

The majority of bites in cats (78.0%) and dogs (80.6%) occurred between 9 a.m. and 6 p.m. Both species had peaks at 10 a.m. and again at 12 p.m., with a decrease between 1 p.m. and 2 p.m. Cat bites peaked at 3 p.m., whereas dog bites remained more consistent between 2 p.m. and 4 p.m. (Figure 4).

**Figure 4:**
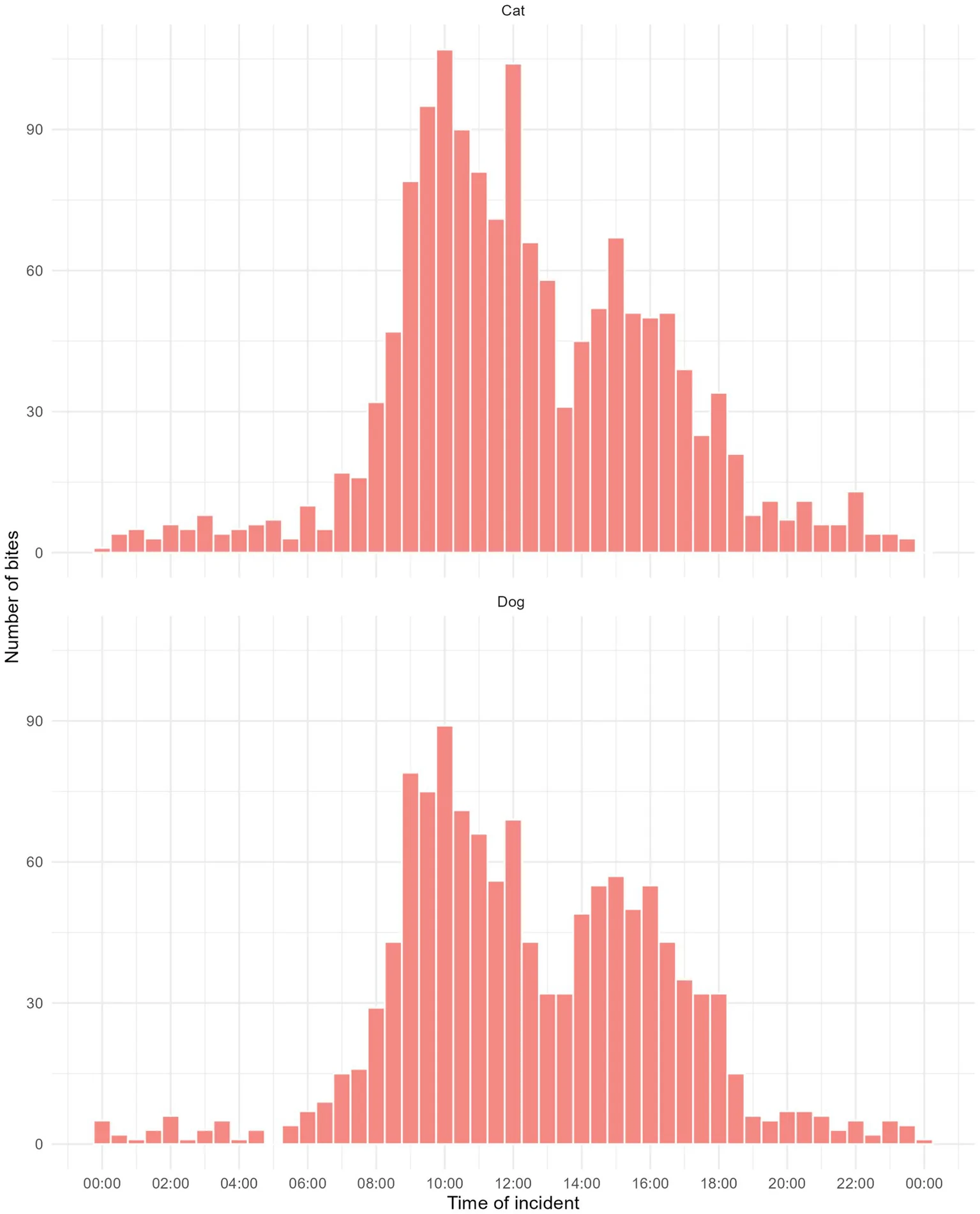
Time of dog (*n* = 907) and cat bite incidents (*n* = 1,057).

### 3.6 Location of bite in clinics

Cat bites occurred most often in the consult room (49.03%), followed by the preparation area (29.85%), kennel area (16.32%), and theatre (4.53%). Dog bites occurred in the consult room 52.76% of the time, followed by the preparation area (25.74%), kennel area (16.96%), and theatre (5.45%). In the consult room, bites occurred most frequently between 9 a.m. and 12 p.m. in both cats and dogs, and both species had afternoon peaks at 3 p.m. in the consult room. Bites were also most frequently experienced in the morning in the preparation room across both species (Figure 5).

**Figure 5:**
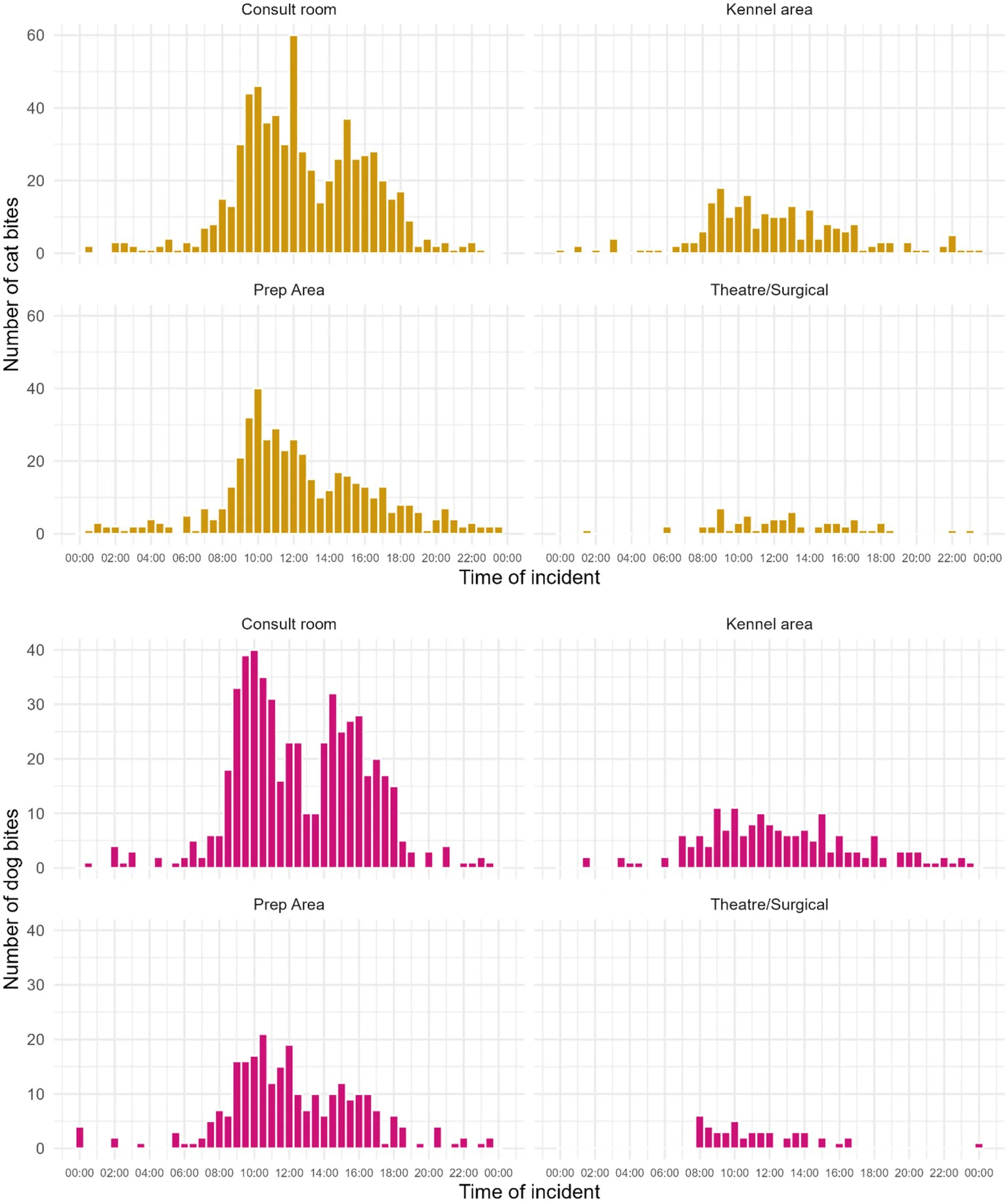
Location in clinic where bite incidents occurred by time [cats (red) (*n* = 1,016); dogs (blue) (*n* = 783)].

### 3.7 Clinic size and bite frequency

We examined if clinics with more registered patients reported more bites. The correlation between the number of registered cats and bite incidents was weak and not statistically significant (*r*_s_ = 0.20, *p* = 0.43). Similarly, the number of registered dogs showed no association with bite frequency (*r*_s_ = 0.02, *p* = 0.95).

### 3.8 Job role

There were a higher proportion of bites reported by veterinary nurses for dog (53.36%; *n* = 484) and cat (63.39%; *n* = 670) bites compared to veterinarians and support staff (Table 2). A significant association was found between job role and species in reported bite incidents (χ^2^ = 20.30; df = 2; *p* < 0.001) (Table 2).

**Table 2:** Proportion of dog and cat bite incidents by professional role.

Professional role	Cat bite (%)	Dog bite (%)
Veterinarian	32.82	41.56
Nurse	63.38	53.36
Support staff	3.78	5.07

Regarding where the bites occurred and if professional role was associated with location in clinic, there was a significant association between job role and the location of cat bite incidents in clinics (χ^2^ = 124, df = 3, *p* < 0.001). Vets experienced more bites in consult rooms, whereas nurses were bitten more frequently in kennels and preparation areas. The same finding was seen in dogs (χ^2^ = 111, df = 3, *p* < 0.001).

## 4 Discussion

This study provides an overview of dog and cat bite incidents reported across a large veterinary chain practises during 2024 and 2025, including species differences, bite locations, timing, and professional role. Across the recorded bite incidents, cats and dogs were responsible for the majority of bites; this is consistent with previous research [e.g., Tulloch et al., and Johnson and Fritschi (4, 8)]. However, this research further examined how bite incidents are described, where and when they occur in clinics, and how they relate to professional roles.

A key finding of this audit was that where emotional descriptors were used, the most common response described both cats and dogs as “aggressive,” rather than using terms that reflect the animals’ underlying emotional states (e.g., fearful). It may indicate the limited use of behaviourally informed terminology or uncertainty in recognising the emotional drivers and motivations for behaviour. This potentially suggests that people perceive aggression as a fixed or inherent trait, rather than a context-dependent behaviour. Previous research supports this assumption, as Tami and Gallagher (18) found that when showed a video of a playful dog biting the muzzle of a younger dog, participants frequently regarded the behaviour as aggressive despite the surrounding context (e.g., first social interaction in a public park, tail wagging, ears forward). This study cannot determine why staff used particular descriptors, but the predominance of the term “aggressive” suggests that incident reporting may describe the outcome of the interaction versus the behavioural context.

From an ethological perspective, no animal is inherently “aggressive”; aggressive behaviours are part of a normal behavioural repertoire used to increase distance and avoid actual or perceived threats and/or physical conflict or pain (19, 20). The term “aggression” is broad and often lacks agreement within and across both the human and animal field (21, 22). Therefore, labelling animals as aggressive risks misinterpreting behaviour and could contribute to pathologising normal behavioural responses. This distinction is not purely semantic; how behaviour is labelled may directly influence handling decisions and may escalate rather than mitigate stress in the animal. In addition to language misinterpretation, pre-existing veterinarian biases may also influence how risk is perceived and managed. For example, Kogan et al. (23) surveyed US veterinarians and highlighted that specific breeds were more frequently perceived to be at a higher risk of a serious bite (e.g., Chow Chow, Chihuahua) in comparison to other moderate (e.g., Doberman) or low risk breeds (e.g., Beagle).

Such biases may be reinforced via limited behaviour training and low perceived self-confidence. Kogan et al. (24) surveyed 1,082 US veterinarians, of which only 43.8% stated that they felt they had received significant training on animal behaviour during their veterinary qualification. Further, research of 95 veterinary professionals in Ireland indicated that a substantial proportion have a low level of confidence regarding feline behaviour problems (25). Catalán et al. (26) also conducted a survey of Chilean veterinarians and found that just over half (57.5%) could correctly recognise non-verbal behavioural cues in dogs, though a higher proportion (66.2%) were able to recognise verbal cues. Clinical experience also played a role in the ability to recognise cues; those with >20 years or more experience were found to be more able to accurately identify behavioural cues in dogs (26). Improving the consistency and specificity of behavioural terminology in veterinary medicine may improve communication between colleagues and facilitate more appropriate handling decisions, thus being more patient-centric and reducing the risk of future bite incidents. If pain, fear, or frustration related behaviours are considered simply as aggression, opportunities to identify modifiable triggers and implement preventative handling strategies may be missed.

The predominance of hand and finger injuries from cat bites reflects that during handling and restraint, cats may respond with defensive bites directed towards the handler’s hands, which are closest to the animal’s head. Similarly, dog bites most frequently involved the hands and fingers during restraint; however, 18% of bites were also to the arms as this may be a more accessible target in particular situations. These findings are consistent with the public (27) and veterinary professionals (15) prior experience, where bites predominantly occur on the hands, fingers, wrists, and arms. For example, Catalán et al. (26) found that self-reported dog bite victims were most commonly bitten due to some form of interaction (stroking, restraining/handling the dog), and the locations where they were most commonly bitten were the hand, wrists and upper extremities [also see Wake et al., and Mills (22, 28)]. Within an occupational context, existing evidence is somewhat consistent with the present study, as dog bites to the upper and lower extremities were the most reported bite locations amongst occupational workers, including both dog (e.g., kennel workers, veterinarians and veterinary nurses) and non-dog professionals (e.g., postal workers) (29). This highlights that human behaviour towards the dog, the dog’s history and behaviour, and the surrounding environment needs careful consideration (30).

Previous research has explored handling during veterinary consultations. For example, recently, Cisneros et al. (31) explored behavioural (e.g., lip licking, yawning vocalising, attempting to escape) and physiological (heart, respiration and body temperature) measures in relation to five different handling and restraint methods, each of increasing intensity. They found that dogs in the most restrictive technique, the full body hold, vocalised significantly more when compared to the least restrictive passive restraint method. They also found that fear scores were significantly lower during the passive restraint compared to all other handling methods. Similarly, low stress handling interventions has also been found to reduce cortisol over four veterinary visits, in comparison to routine handling procedures (32). This suggests that more restrictive handling methods may increase stress-related behaviours, which could increase the likelihood of a bite. Initiatives like the dog [e.g., Dog Trust (33)] and cat friendly clinic schemes [e.g., International Cat Care (34)], which aim to reduce the stress experience in the veterinary clinic, may therefore prove impactful for reducing bite risk in-clinic.

Regarding time of day the incident occurred, both species showed a clear bimodal trend, with most bites occurring between 9 a.m. and 6 p.m., with peaks around 10 a.m., 12 p.m., and 3 p.m. These results are consistent with previous research exploring injuries to companion animal staff and clients at UK veterinary practises (9). These times correspond to typical periods of high clinical activity, suggesting that workload intensity may influence the risk of bites. The peak around midday may be related to workflow during busy consultation periods. However, because staffing levels, appointment schedules and workload were not measured, the reasons cannot be determined from this audit. Interestingly, although increased bite frequency appeared to correlate against times of greater case throughput within a clinic, we did not find a significant relationship between the number of registered cats and dogs and bite report frequency. This suggests that bite risk may not be intrinsically influenced by patient load on a clinic population level, but instead that other human-related factors such as staff experience and communication skills, human animal interactions (i.e., handling techniques), and the clinic environment may be more likely to have an influential role in bite incidents. It may also be that the number of registered patients is not a good proxy for workload, as it does not account for consultation frequency, case complexity, staffing levels, or the number of animal handling interactions. Studies incorporating measures of clinical workload alongside human factors are needed to better understand the determinants of bite incidents.

The location data further demonstrates that most bites occurred in consult rooms, followed by preparation and kennel areas, similar to previous research (4). Veterinary surgeons were more likely to be bitten in consult rooms whereas nurses were more often bitten in kennels and preparation areas. This variation in bite frequency based on location in clinic may suggest that for bites and other injuries, prevention strategies may need to be tailored not only to species but also to the specific clinical tasks and environments associated with different roles. Veterinary surgeons frequently perform physical examinations and procedures within consult rooms and nurses may more frequently handle hospitalised patients in wards and preparation areas, including examination, administering treatments, and undertaking procedures. These specific interactions may increase the likelihood of contact with animals experiencing pain, fear, anxiety, or frustration, all of which have the potential to contribute to defensive and protective behaviour. From a safety perspective, a greater emphasis on behavioural assessment prior to handling, communication of identified risks between team members, and the implementation of species-appropriate, low-stress handling approaches may help to prevent or mitigate the risk of bites (35).

This audit was based on retrospective incident reports, which are inherently limited by the accuracy and completeness of the information recorded at the time of the report. As the data are cross-sectional, this prevents any evaluation of causality. To capture all injury types and severities, specific definition of injuries was not included, such as dog bites and therefore may be subjective (e.g., what may constitute a dog bite may vary [see Oxley et al. (10)]). Although, it could be assumed that the reporting of an injury would be sufficient to include a significant injury, for example, a dog bite may result in break to the skin. Further work to update and improve data reporting would be useful to ensure more accurate data. Additionally, these data do not capture longer-term outcomes following bite incidents, including medical treatment given, longer term psychological impact, or changes in working practises within-clinic following a bite incident. The data in this study broadly cover the United Kingdom and Ireland but does not include a breakdown by geographical location. This is an area that could be incorporated into future work to identify injuries and how these relate to local and national demographic factors. These data are from a single veterinary group within the UK, which may limit generalisability to all UK small animal clinics.

Future prospective studies could address these limitations by collecting standardised data at the time of each incident, including contextual factors such as the animal’s history, signs indicative of pain, fear anxiety or stress, and the specific procedures being performed. Incorporating measures of handling method used, staff confidence in handling and the understanding of animal behaviour would also provide qualitative insight into human factors contributing to bites. A more comprehensive understanding of both animal and human-related factors is vital for developing effective, evidence-based strategies to reduce bite risks and improve safety within veterinary clinics.

## Data Availability

The data analyzed in this study is subject to the following licences/restrictions: Self-reported health and safety data restricted to IVC Evidensia employees. Requests to access these datasets should be directed to rachel.malkani@ivcevidensia.com.
