# Supplementary material for: Epidemiology of voluntarily reported dog and cat bite incidents in a large-scale, multi-site veterinary network in the UK and Ireland
Source: Front Vet Sci. 2026 Oct 6;13:1916141. doi: 10.3389/fvets.2026.1916141 (PMC13680216; doi:10.3389/fvets.2026.1916141)
Supplement: Supplementary file 1 [file Table_1.DOCX]

**Appendix 1**

Audit of voluntarily reported dog and cat bite data in IVC Evidensia clinics located in the UK and Ireland Appendices

Reporting questions

Incident Severity

Date of Incident

Time of Incident

Exact Location

Where did the incident happen?

Describe the incident as fully as possible in your own words

Who Was Involved

Was a Person Involved?

Was Injury Or Ill Health Sustained?

Near Miss?

Lost Time Accident?

Is the incident reportable to Health and Safety authorities?

Incident Type

Which category best describes the incident?

If the injury was to a member of the public, were they taken directly to hospital?

What treatment was provided and by whom?

Is a Full Investigation Required?

Recommendations/Preventative Actions

Org Unit

Occupation / Job Type

Persons working hours

Job Family

Animal Involved?

Animal Type

What type of work was being carried out during the incident?

Was the Incident work related

**Appendix 2**

Anatomical location of bite

Dog

```
Part.s..Of.Body.Affected count percent total_n
   <chr>                    <int>   <dbl>   <int>
 1 Hand                       146   38.0      384
 2 Finger/s                    96   25        384
 3 Arm                         70   18.2      384
 4 Other Parts Of Face         21    5.47     384
 5 Leg                         16    4.17     384
 6 Wrist                        8    2.08     384
 7 Eye                          6    1.56     384
 8 Right side of face           6    1.56     384
 9 Head (Excluding Face)        5    1.3      384
10 Torso                        3    0.78     384
11 Neck                         2    0.52     384
12 Unspecified Locations        2    0.52     384
13 Ear                          1    0.26     384
14 Elbow                        1    0.26     384
15 Knee                         1    0.26     384
```

Cat

```
Part.s..Of.Body.Affected count percent total_n
   <chr>                    <int>   <dbl>   <int>
 1 Hand                       194   44.8      433
 2 Finger/s                   155   35.8      433
 3 Arm                         51   11.8      433
 4 Wrist                       16    3.7      433
 5 Other Parts Of Face          3    0.69     433
 6 Eye                          2    0.46     433
 7 Several Locations            2    0.46     433
 8 Unspecified Locations        2    0.46     433
 9 Back                         1    0.23     433
10 Foot                         1    0.23     433
11 Head (Excluding Face)        1    0.23     433
12 Knee                         1    0.23     433
13 Leg                          1    0.23     433
14 Rib                          1    0.23     433
15 Right side of face           1    0.23     433
16 Shoulder                     1    0.23     433
```
